# The Immunosuppressant Fingolimod (FTY720) for the Treatment of Mechanical Force-Induced Abnormal Scars

**DOI:** 10.1155/2020/7057195

**Published:** 2020-01-07

**Authors:** Masayo Aoki, Akatsuki Kondo, Noriko Matsunaga, Azusa Honda, Yuri Okubo, Kazuaki Takabe, Rei Ogawa

**Affiliations:** ^1^Department of Plastic, Reconstructive and Aesthetic Surgery, Nippon Medical School, Tokyo, Japan; ^2^Depertment of Biochemistry and Molecular Biology, Nippon Medical School, Tokyo, Japan; ^3^Division of Breast Surgery, Department of Surgical Oncology, Roswell Park Comprehensive Cancer Center, Buffalo, NY, USA; ^4^Department of Surgery, University at Buffalo Jacob School of Medicine and Biomedical Sciences, The State University of New York, Buffalo, NY, USA

## Abstract

**Aim:**

Abnormal scars such as hypertrophic scars (HSs) and keloids are excessively growing scars that exhibit chronic inflammation and capillary vasculogenesis. The lipid mediator sphingosine-1-phosphate (S1P) is important in inflammatory cell recruitment and angiogenesis. Fingolimod (FTY720) is an analog of S1P and thus functionally antagonizes S1P receptors and inhibits the enzyme that produces S1P. We examined the effects of topical FTY720 injections on mechanical force-induced HS progression.

**Methods:**

Mechanical force-induced HSs were generated in C57BL6/J mice by suturing a dorsal incision and applying a stretching device on Days 6, 8, 10, and 12. On Days 8, 10, and 12, intracutaneous FTY720 (10 *μ*M) or control vehicle injections were performed. On Day 14, scar tissues and blood were procured and subjected to histology and flow cytometry.

**Results:**

Flow cytometry showed that FTY720 decreased the frequencies of macrophages with M2 predominance in the scars but had no effect on total, CD4^+^, or CD8a^+^ T cell frequencies. FTY720 also decreased the vascular endothelial cell frequencies in the scar along with the microvessels, as determined by immunohistochemistry. Compared to the vehicles, FTY720 treatment significantly reduced the gross scar area and the cross-sectional scar area on histology. On the other hand, FTY720 tended to reduce white blood cells and significantly reduced the lymphocyte frequencies in the blood.

**Conclusion:**

Topical FTY720 induces M2 predominance and impairs angiogenesis. Therefore, its local immunosuppressive mechanisms differ from those of conventional immunosuppressive agents. Topical FTY720 can be a novel therapeutic option for abnormal scars that are difficult to control with corticosteroids. Its lymphocytopenic effects may be limited by careful optimization of the treatment regimen.

## 1. Introduction

Human hypertrophic scars (HSs) and keloids are fibroproliferative disorders of the skin that arise after wounding that impairs cutaneous integrity and induces deep dermal destruction. The wounds may be generated by surgical procedures, burns, infections, or other causes of damage to the deep dermis. Both scar types are characterized by chronic inflammation and vasculogenesis [1]. Abnormal scars such as HSs and keloids often lead to contractures, symptoms such as pain or itch, cosmetic issues, and lifelong disability [2]. The pathogenesis of these abnormal scars involves repetitive or continuous mechanical stress [3]. This stress helps to initiate and then enhance inflammation. This leads to ceaseless inflammation in the scar. The inflammation is also localized; it is mainly found in the reticular layer of the dermis of the skin [4]. The proangiogenic and fibroproliferative effects of the inflammation explain why the reticular layer of abnormal scars displays substantial vasculogenesis and collagen accumulation [5].

At present, the mainstream of conservative treatment for abnormal scars is the topical application or injection of corticosteroids. However, severe keloids may require surgical resection followed by radiotherapy [6]. In recent years, laser irradiation or botulinum toxin has also been used [7, 8]. However, it takes a very long time before these treatments convert abnormal scars into mature and soft scars, and it is sometimes difficult to control with such conventional treatment strategies in severe cases [9].

These limitations indicate that more therapeutic options or effective approaches are needed. Given the pathogenic mechanisms driving abnormal scar formation and progression, it may be that combination therapy with multiple bioactive compounds that suppress inflammation, vasculogenesis, fibrosis, and/or mechanical stress will be particularly effective for the management of abnormal scars.

One such bioactive compound may be FTY720 (also known as fingolimod/Gilenya), which was originally discovered by chemical modification of myriocin, a natural product. It is an analog of the lipid mediator sphingosine-1-phosphate (S1P), which regulates the proliferation, survival, and migration of mammalian cells [10], is essential for vascular development, neurogenesis, and lymphocyte trafficking [11–13], and acts as a second messenger during inflammation [14]. S1P is generated from ceramide in two steps: first, ceramidase converts ceramide into sphingosine, after which sphingosine kinases convert sphingosine to S1P [15]. S1P exerts its cellular functions by both intracellular mechanisms and *via* extracellular receptors called S1P receptors (S1PRs). FTY720 is both a functional antagonist of four S1PRs except S1PR2 and inhibits sphingosine kinase 1. FTY720 (and other S1PR-modifying compounds) thus effectively block the recruitment of various types of inflammatory cells, including lymphocytes, macrophages, and monocytes, to inflamed sites [16, 17]. As a result, FTY720 is a potent immunosuppressant. Indeed, in 2018, it was approved as a first-line therapy for relapsing forms of multiple sclerosis [18]. Moreover, the use of 0.5, 2.5, or 5 mg FTY720/day in kidney transplant patients decreases the number of T and B cells in the circulation [19]. Persistent FTY720 therapy in MS patients also significantly reduces the circulating numbers of an NK cell subpopulation (CD56^bright^). In addition, FTY720 treatment alters the chemokine receptivity profile of NK cells from MS patients [20]. Moreover, FTY720 suppresses the cytotoxic functions of CD8^+^ T cells; however, this effect is not due to the ability of FTY720 to modulate S1P signaling [21]. Notably, oral FTY720 administration in mice strongly blocked vascular endothelial growth factor-induced vascular permeability *in vivo* [22]. It may also suppress angiogenesis [23]. Moreover, local administration of FTY720 ameliorates several other inflammatory diseases, including allergic asthma, allergic conjunctivitis, and allergic contact dermatitis [24–27].

Given the key role that inflammation plays in the development and progression of abnormal scars and the fact that FTY720 suppresses inflammatory cell recruitment, we hypothesized that topically applying FTY720 to heavy scars may decrease the inflammatory cell numbers in the scar and inhibit angiogenesis. To test this hypothesis, we examined the effects of topical FTY720 injection on HS-like scars in mice that were induced with mechanical force.

## 2. Materials and Methods

### 2.1. Mice and Mechanical Load-Induced Hypertrophic Scar Model

All animal procedures were approved by the Animal Experimental Ethical Review Committee of Nippon Medical School and were performed according to the institutional guidelines for animal care (Nippon Medical School, Tokyo, Japan). Eight-week-old male C57BL6/J mice were purchased from Tokyo Experimental Animals Supply Co. (Tokyo, Japan). The hypertrophic scar model was generated with biomechanical loading induced by VECTOR 620 expansion screws (SCHEU, Iserlohn, Germany) as previously described [28, 29]. Briefly, a 2 cm long (head side) and 1 cm long (caudal side) full-thickness incision (1 cm apart) created on the dorsal midline of each mouse was sutured with 4-0 VICRYL (Johnson & Johnson, New Brunswick, NJ). The sutures were removed on Day 6 after incision. The mechanical loading devices were placed over the head side scars and sutured to the skin on either side of the incision with 5-0 ETHILON (Johnson & Johnson). Sustainable stretching was optimized on Days 6, 8, 10, and 12. The caudal side scars were nonloading negative control scars to confirm whether mechanical loading is working.

### 2.2. FTY720 Preparation and Topical Injection in Mice

FTY720 was purchased from Cayman Chemicals (Ann Arbor, MI). FTY720 powder was dissolved in ethanol to a 50 mM stock solution. Ethanol alone (control vehicle solution) and the 50 mM FTY720 stock solution were diluted with saline by 5000-fold. Thus, the diluted FTY720 solution had a concentration of 10 *μ*M. The vehicle and 10 *μ*M FTY720 solutions were then sonicated to make the lipid uniform in saline. Mice were divided into two groups on postincision Day 8, and the mechanical loaded scars of one group were intracutaneously injected with 200 *μ*l of vehicle solution while those of the second group were injected with 200 *μ*l of FTY720 in same way. The each nonloading negative control scars were injected with 100 *μ*l of the same solutions. The injections were repeated on Days 10 and 12. Thus, the mice were injected with 51.6 *μ*g FTY720/kg/day. This dose was determined to be effective in terms of reducing scarring by pilot studies (data not shown). The scar tissues and blood were procured on Day 14 for analysis. Whether FTY720 induced lymphocytopenia was assessed by whole blood counting and white blood cell differentiation.

### 2.3. Cell Culture

The RAW264.7 macrophages were purchased from KAC (Kyoto, Japan) and cultured in Dulbecco's Modified Eagle's Medium supplemented with 2 mM glutamine and 10% fetal bovine serum (FBS). Lipopolysaccharide (LPS) from E. coli O111 was purchased from Fujifilm Wako (Osaka, Japan). The cells were plated in a 24-well plate at densities of 5 × 10^4^ cells/well and were grown until subconfluency. After cells were culture for 3 hours in serum-free medium, cells were cultured with vehicle or 2 *μ*M FTY720 with 100 ng/ml LPS for 24 hours. The cells were then harvested for RNA extraction and flow cytometry.

### 2.4. Flow Cytometry

Cells were isolated from the scar tissues as described previously [30]. Thus, the tissues were cut into small pieces and digested at 37°C for 90 min in DMEM containing 10% FBS, 1.2 mg/ml hyaluronidase (Sigma-Aldrich), 2 mg/ml collagenase (Sigma-Aldrich), and 0.2 mg/ml DNase I (Sigma-Aldrich). The cell pellets were resuspended in flow cytometry buffer (PBS containing 2% FBS and 2 mM EDTA). RAW264.7 macrophages were trypsinized and resuspended with flow cytometry buffer. Cells were incubated with the anti-CD16/32 antibody for 5 min to block the Fc*γ* receptors. To measure inflammatory cell recruitment, the scar cell preparations were stained with fluorescein isothiocyanate- (FITC-) conjugated anti-CD3, allophycocyanin- (APC-) conjugated anti-CD4, brilliant violet (BV) 510-conjugated anti-CD8a, and phycoerythrin- (PE-) conjugated anti-F4/80 antibodies at 4°C for 20 min. Then, cells were fixed and permeabilized using the BD Cytofix/Cytoperm Fixation/Permeabilization Kit and labeled with an Alexa Fluor 647-conjugated anti-CD206 antibody. M1/M2 polarization was analyzed as described previously [31]. To measure angiogenesis, the cells were stained with APC/Cy7-conjugated anti-CD45 and PE/CY7-conjugated anti-CD31 antibodies. The cells were analyzed with FACSVerse and FACSuite software (BD, San Jose, CA). All antibodies were purchased from BD.

### 2.5. Immunohistochemistry

Paraffin-embedded sections were stained with H&E and a primary antibody against CD34 (Abcam, Cambridge, UK). The immunostained sections were developed with VECTASTAIN Universal Elite ABC Kit (Vector, Burlingame, CA).

### 2.6. Gross Scar Area Analysis

The scars were photographed at the indicated time points. The digital photos were analyzed using GIMP 2.8 software. The pixels of the scar area were normalized to the pixels of the same scale.

### 2.7. Histological Analysis of the Scars

On Day 14, the mice were bled and euthanized by isoflurane. The scars were resected, embedded in paraffin, and sectioned in cross section. The sections were then stained with H&E and Masson's trichrome and photographed under low and high magnification. The cross-sectional area of the scars, scar elevation index (SEI), collagen area, and collagen integrated density were determined as described previously using GIMP2.8 and ImageJ imaging analysis [32].

### 2.8. Quantitative RT-PCR

Total RNA was extracted by using RNeasy Mini Kits (Qiagen, Valencia, CA) according to the manufacturer's recommendations. cDNA was then synthesized by using High-Capacity cDNA Reverse Transcription Kits (Applied Biosystems, Foster City, CA). qRT-PCR was performed by using an ABI Prism 7500 System (Applied Biosystems) with Power SYBR Green master mix (Applied Biosystems). For each primer set, the optimal dilution was determined and melting curves were used to determine the amplification specificity. Gapdh served as the internal control. The primer pairs used are as follows: Gapdh, forward, 5′-AGGTCGGTGTGAACGGATTTG-3′, and reverse, 5′-TGTAGACCATGTAGTTGAGGTCA-3′; IL6, forward, 5′-GCTTAATTACACATGTTCTCTGGGAAA-3′, and reverse, 5′-CAAGTGCATCATCGTTGTTCATAC-3′; Tnf, forward, 5′-ACTGAACTTCGGGGTGATCG-3′, and reverse, 5′-CCACTTGGTGGTTTGTGAGTG-3′; Mrc1, forward, 5′-TTGCACTTTGAGGGAAGCGA-3′, and reverse, 5′-CCTTGCCTGATGCCAGGTTA-3′; Arg1, forward, 5′-TCATGGAAGTGAACCCAACTCTTG-3′, and reverse, 5′-TCAGTCCCTGGCTTATGGTTACC-3′; and IL10, forward, 5′-GCTCTTACTGACTGGCATGAG-3′, and reverse, 5′-CGCAGCTCTAGGAGCATGTG-3′. Relative expression was calculated by using the 2^–*ΔΔ*Ct^ method with correction for different amplification efficiencies.

### 2.9. Enzyme-Linked Immunosorbent Assay (ELISA)

The levels of interleukin- (IL-) 6 and IL-10 in serum were measured with the ELISA MAX standard sets (BioLegend, San Diego, CA) according to the protocol's instructions.

### 2.10. Quantification of FTY720 by Mass Spectrometry

Lipids were extracted from serum and tissues, and sphingolipids were quantified by liquid chromatography-electrospray ionization-tandem mass spectrometry (LC-ESI-MS/MS; 5500 QTrap, AB Sciex, Framingham, MA) as described [33].

### 2.11. Statistical Analysis

The vehicle and FTY720 groups were compared in terms of continuous variables by using a Wilcoxon signed-rank sum test, Student's *t*-test, or Welch's *t*-test after *F* test. *p* < 0.05 was considered significant. All statistical analyses were performed using EZR (Jichi Medical University, Japan).

## 3. Results

### 3.1. Topical Injection of FTY720 Did Not Influence Lymphocyte Recruitment into the Scar

**Figure 1:**
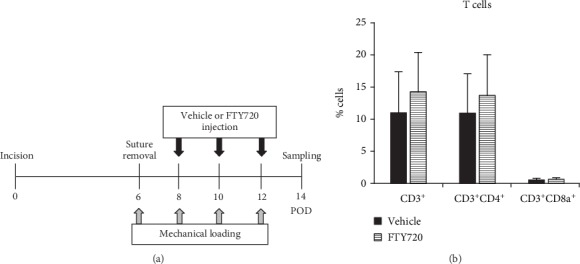
Effect of topical FTY720 injection on the Day 14 T cell population in the scars. (a) Schematic depiction of the schedule in which mechanical force-induced hypertrophic scars were generated in mice and treated with FTY720 injections. (b) The frequencies of CD3^+^, CD3^+^CD4^+^, and CD3^+^CD8a^+^ T cells in the scars were determined by flow cytometry (*n* = 7). Two independent experiments were performed. Both showed similar results. All values shown represent means ± s.e.m.

We injected the developing murine HSs with FTY720 during the proliferative stage of scar formation (postincision Days 8, 10, and 12), and the scars and blood were procured on Day 14. The flow of mechanical stress-induced HS formation and FTY720 injections in the mice is shown in Figure 1(a). S1P is essential for lymphocyte trafficking and promotes the trafficking of these cells to inflamed tissues [24]. FTY720 induces the retention of lymphocytes in lymph nodes, thereby preventing them from contributing to inflammation in other areas of the body [24]. These observations led us to expect that local injection of FTY720 would reduce the number of lymphocytes in the scars. However, when we analyzed the T cell population in the scars with flow cytometry, we found that FTY720 did not reduce either the total (CD3^+^), helper (CD3^+^CD4^+^), or cytotoxic (CD3^+^CD8a^+^) T cell populations in the scars (Figure 1(b)).

### 3.2. Topical Injection of FTY720 Reduced the Number of M1 Macrophages in the Scars

**Figure 2:**
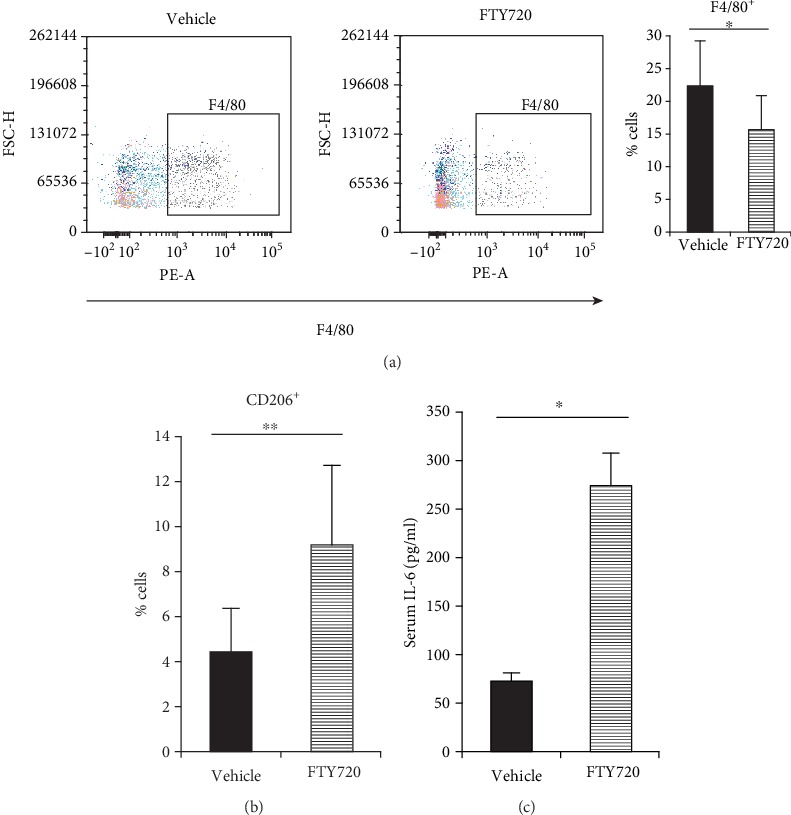
Effect of topical FTY720 injection on Day 14 scar macrophage frequencies and M1/M2 polarization. (a) Representative flow cytometric plots are shown along with the average frequencies of F4/80^+^ cells. (b) The percentages of CD206^+^ cells were determined by flow cytometry (*n* = 7). Two independent experiments were performed. Both showed similar results. (c) The level of serum IL-6 was determined by ELISA (*n* = 4). All values shown represent means ± s.e.m.^∗^*p* < 0.05 and ^∗∗^*p* < 0.01, as determined by Student's *t*-test or Welch's *t*-test after *F* test.

Next, we analyzed the macrophages in the Day 14 mechanical stress-induced HSs by using flow cytometry. Compared to the vehicle group, the FTY720 group had significantly smaller frequencies of F4/80^+^ cells, which are considered to be macrophages [31] (Figure 2(a)). However, a closer analysis of the M1 and M2 macrophages in the F4/80^+^ cell population showed that FTY720 significantly increased the frequencies of CD206^+^ cells (Figure 2(b)). CD206^+^ cells are considered to be M2-polarized macrophages [31]. We measured serum cytokine levels to evaluate the systemic effects of local FTY720 injection on macrophage polarization. In contrast to local reactions, a serum IL-6 level was significantly increased in mice injected with FTY720 compared with mice injected with vehicle (Figure 2(c)). Serum IL-10 was not detected in all serum samples.

### 3.3. Topical Injection of FTY720 Inhibited Angiogenesis in the Scars

**Figure 3:**
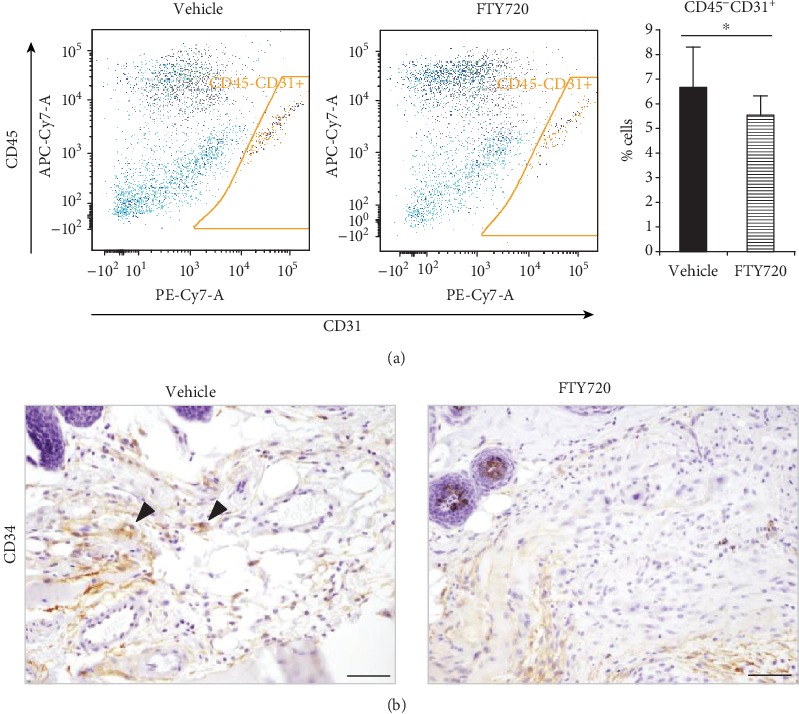
Effect of topical FTY720 injection on angiogenesis in the Day 14 scar. (a) Representative flow cytometric plots and the average frequencies of CD45^−^CD31^+^ cells in the scars were determined by flow cytometry (*n* = 14). Two independent experiments were performed. Both results were aggregated. (b) CD34 immunostaining images of the scars on Day 14. Arrowheads show CD34^+^ microvessels (scale bars: 50 *μ*m). All values shown represent the means ± s.e.m.^∗^*p* < 0.05, as determined by Student's *t*-test or Welch's *t*-test after *F* test.

Since S1PR1 signaling is essential for angiogenesis [34], we examined the effect of local FTY720 injection on angiogenesis in the scars by flow cytometry and immunohistochemistry. The FTY720 injections significantly decreased the frequency of CD45^−^CD31^+^ cells in the scars; these cells are considered to be vascular endothelial cells (Figure 3(a)). Furthermore, while the immunohistochemical analyses showed the presence of several CD34^+^ capillaries in the vehicle-treated scars, these capillaries were not observed in any of the FTY720-treated scars (Figure 3(b)).

### 3.4. Topical Injection of FTY720 Reduced the Gross Scar Size

**Figure 4:**
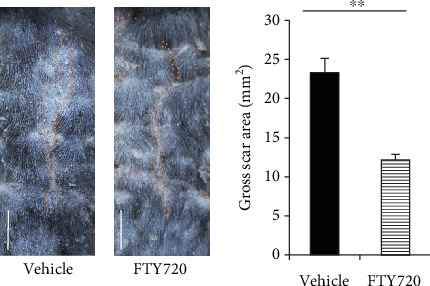
Effect of topical FTY720 injection on the gross scar area. Representative images of the gross scar area (dotted line) on Day 14 are shown. The gross scar areas on Day 14 were plotted (*n* = 15). Five independent experiments were performed. Both showed similar results. The values shown represent means ± s.e.m.^∗∗^*p* < 0.01, as determined by Wilcoxon signed-rank sum test.

In our preliminary experiments, topical injection with 5 *μ*M FTY had little effect on the gross scar size. The 10 *μ*M FTY720-treated mice displayed significantly less scar formation than the vehicle group, as indicated by the lower average gross scar area on Day 14 (Figure 4).

### 3.5. Topical Injection of FTY720 Reduced Histological Hypertrophic Scar Formation

**Figure 5:**
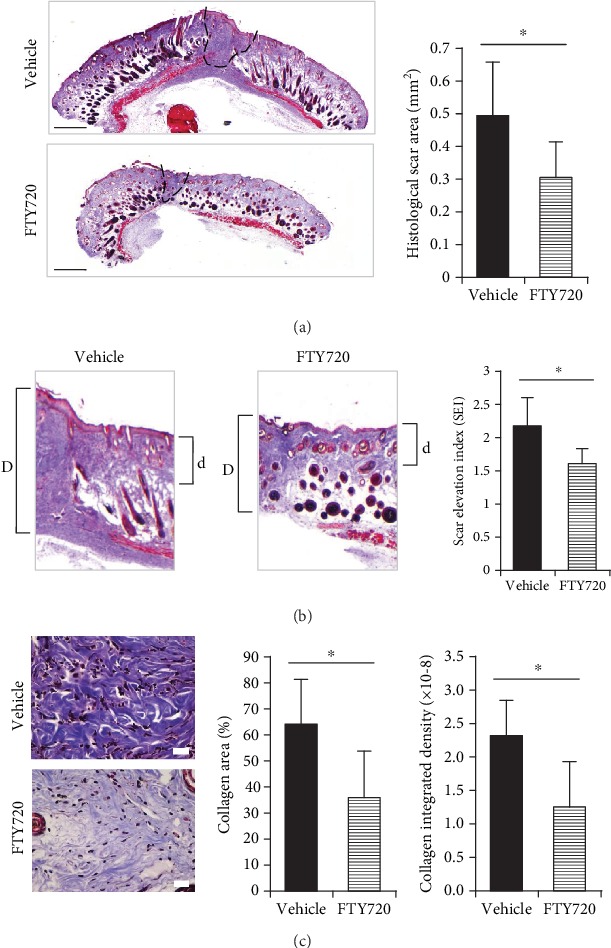
Histological analysis of the effect of topical FTY720 injection on Day 14 cross-sectional scar formation. (a) Representative Masson's trichrome stain images of the histological scar area (dotted line) are shown. The average cross-sectional scar areas were plotted (scale bars: 500 *μ*m, *n* = 7). (b) Quantification of scar elevation index (SEI). “*D*” indicates scar thickness, and “*d*” indicates adjacent normal skin thickness. SEI is defined by the *D*/*d* ratio (*n* = 7). (c) Representative images under high magnification and quantification of the percentage of collagen area and collagen integrated density (scale bars: 20 *μ*m, *n* = 7). Two independent experiments were performed. Both showed similar results. The values shown represent means ± s.e.m.^∗^*p* < 0.05, as determined by Student's *t*-test or Welch's *t*-test after *F* test.

Compared to the vehicle group, the FTY720-treated mice also had significantly smaller scar cross-sectional areas on Day 14, as shown by the histological analysis (Figure 5(a)). The FTY720-treated mice displayed significantly less SEI than the vehicle group (Figure 5(b)). Further, the FTY720-treated scars showed significantly less percentage of the area and integrated density of collagen (Figure 5(c)).

### 3.6. Local Injections of FTY720 Led to Blood Lymphocytopenia

**Figure 6:**
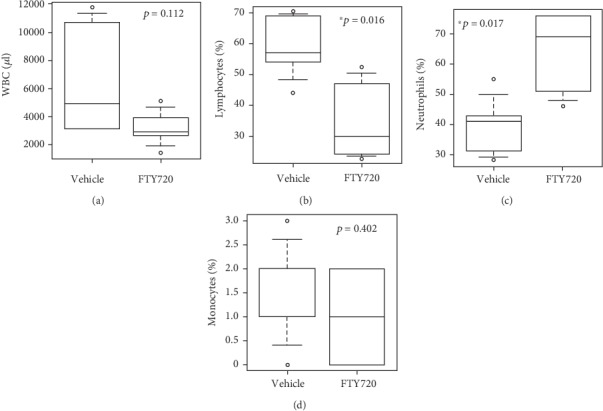
The effects of topical FTY720 injection on the number of total white blood cells and white blood cell types on Day 14. (a) The number of white blood cells, as determined by total blood cell counting. The frequencies of lymphocytes (b), neutrophils (c), and monocytes (d) in the blood. All values shown represent means ± s.e.m. (*n* = 7). Two independent experiments were performed. Both showed similar results. ^∗^*p* < 0.05, as determined by Student's *t*-test or Welch's *t*-test after *F* test.

It is known that systemic administration of FTY720 induces blood lymphocytopenia [35]. To determine whether local FTY720 administration also induces lymphocytopenia and/or other effects, we collected blood from each mouse before euthanization on Day 14 and counted the lymphocytes. The FTY720 group tended to have fewer white blood cells in their circulation overall than the vehicle group (*p* = 0.112). This difference became significant when specific lymphocyte counts were assessed; in particular, the FTY720-treated mice had significantly lower lymphocyte counts (*p* = 0.016) and significantly higher neutrophil counts (*p* = 0.017) in their circulation than the vehicle-treated mice. The two groups did not differ significantly in terms of circulating monocyte frequencies (*p* = 0.402) (Figures 6(a)–6(d)). Thus, even though the injected FTY720 dose was small (51.6 *μ*g/kg/day), the three injections every other day lowered the circulating lymphocyte numbers. These observations suggest that even local injections with small doses of FTY720 may induce the lymphocytopenia. The levels of FTY720 and FTY720-P in scar tissues determined by mass spectrometry were 0.074 ± 0.002 and 0.382 ± 0.072 pmol/mg (average ± SD) on Day 14, respectively (*n* = 3). Serum FTY720 was not detected in any sample.

### 3.7. FTY720 Directly Affects Macrophage Polarization in RAW264.7 Macrophages

**Figure 7:**
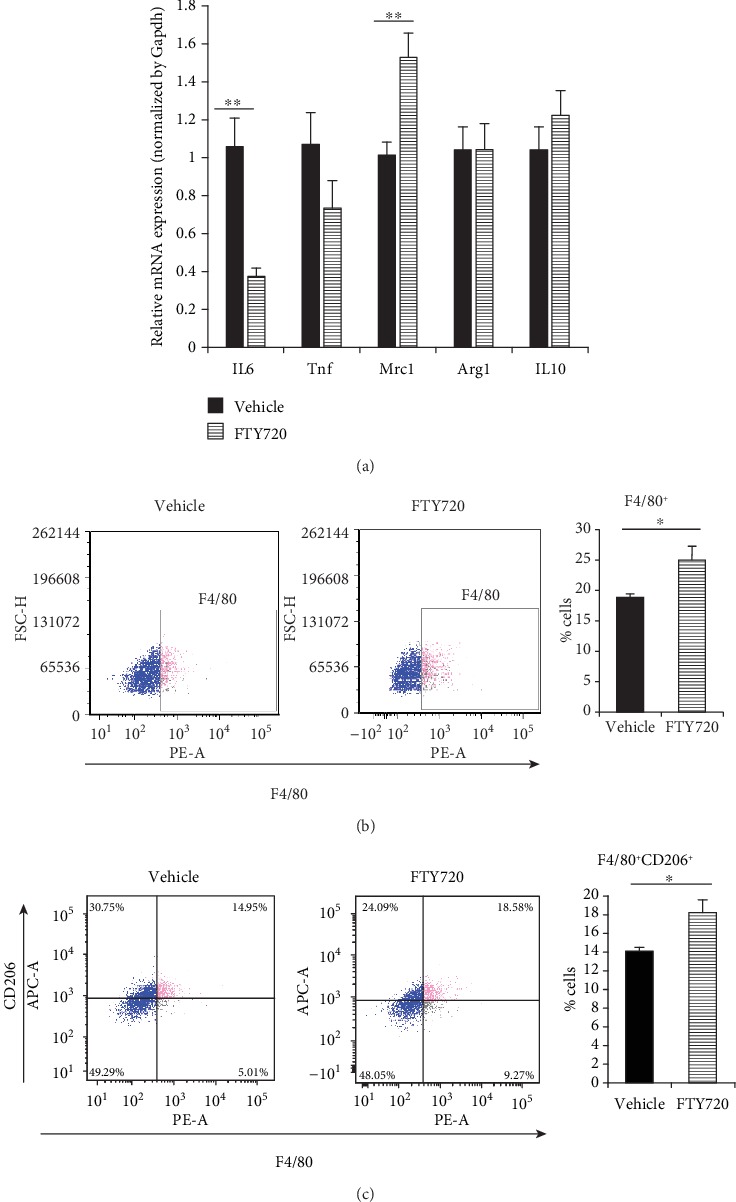
The effect of 2 *μ*M FTY720 with 100 ng/ml LPS in RAW264.7 macrophages. (a) The expression of M1 and M2 marker genes was determined by qRT-PCR (*n* = 6). (b) Representative flow cytometric plots are shown along with the average frequencies of F4/80^+^ cells. The percentages of F4/80^+^ cells were determined by flow cytometry (*n* = 6). (c) Representative flow cytometric plots are shown along with the average frequencies of F4/80^+^CD206^+^ cells. The percentages of F4/80^+^ and F4/80^+^CD206^+^ cells were determined by flow cytometry (*n* = 6). Two independent experiments were performed. Both showed similar results. ^∗^*p* < 0.05, as determined by Student's *t*-test or Welch's *t*-test after *F* test.

We examined whether the effect of topical FTY720 injection on local macrophage polarization is a direct effect. First, we investigated expression levels of IL6 and tumor necrosis factor (Tnf) as M1 marker genes and mannose receptor C type 1 (Mrc1), arginase 1 (Arg1), and IL10 as M2 marker genes [36, 37]. In macrophages activated by LPS stimulation, FTY720 significantly decreased expression of IL6 and increased expression of Mrc1, which is also known as CD206 (Figure 7(a)). FTY720 directly induced macrophages to inhibit M1 polarization and promote M2 polarization. FTY720-induced increases in both the population of F4/80^+^-activated macrophages and the percentage of F4/80^+^CD206^+^ M2 macrophages were observed in flow cytometry (Figures 7(b) and 7(c)).

## 4. Discussion

Studies with the S1P analog FTY720 show that S1P participates in the egress of lymphocytes from the thymus and secondary lymph organs into the circulation [38]. In fact, S1P is indispensable for lymphocyte trafficking and therefore systemic FTY720 treatments result in lymphocytopenia [39]. Given these observations, we predicted that the local administration of FTY720 would reduce the recruitment of lymphocytes to the HS bed, thereby converting the pathogenic scars into mature scars. However, while we did find that local FTY720 injections significantly suppressed pathogenic scar growth, this effect did not associate with decreased total, CD4^+^, or CD8a^+^ T cell frequencies. Instead, we found that local FTY720 injections decreased the total macrophage frequencies in the scars. We also found that local FTY720 treatment had disparate effects on other cell types. Specifically, it markedly reduced the total macrophage frequencies while increasing the M2 macrophage frequencies. It is interesting that the main effect of systemic administration of FTY720 is lymphocytopenia [35] whereas the effect of local application is to decrease the local macrophage frequencies without influencing lymphocyte counts. However, the effects of local administration of FTY720 on lymphocyte activation in inflamed tissues are unclear. In the future, a more detailed investigation of lymphocyte activation would be beneficial for elucidating an expanded indication for local FTY720 administration.

Many studies have shown that macrophages play important roles in scar biology [40]. In the early stages of wound healing, monocytes/macrophages are surrounded by inflammatory stimuli such as LPS and cytokines and differentiate into the “classic” inflammatory M1 phenotype that debrides the wound, produces reactive oxygen species, and secretes inflammatory cytokines that promote beneficial inflammatory responses [41]. Later in wound healing, macrophages differentiate into M2 macrophages that dampen inflammation and promote tissue remodeling [42]. If this switch between M1 and M2 cell populations does not occur, poor wound healing ensues [43]. This is supported by the study of Guo et al., who showed that the poor wound healing in diabetes is driven by harmful glycation products called advanced glycation endproducts, which upregulate macrophage autophagy by activating interferon reguratory factor 8(IRF8); this in turn promotes M1 differentiation. The excessive autophagic activities of the M1 macrophages ultimately impair wound healing [44]. The proinflammatory properties of M1 macrophages also suggest that prolonged M1 differentiation may promote the strong chronic inflammation that characterizes abnormal scars. Thus, regulating macrophage polarization by targeting potential macrophage regulators could improve wound healing, even under conditions that normally yield abnormal fibroproliferative scars. This notion is supported by the present study, which suggests that FTY720 may promote normal wound healing under HS-inducing mechanical stress by increasing M2 polarization during the proliferative stage.

The results of the present study show that FTY720 directly acted on RAW264.7 macrophages by polarizing them, thereby changing them into activated M2 macrophages. Local injection of FTY720 resulted in local polarization to M2 macrophages and increasing serum IL-6 levels. This systemic change may be a secondary response to immunosuppression. FTY720 has previously been reported to increase the numbers of M2 macrophages in remodeling tissues [45, 46]. Qin et al. reported that FTY720 is effective in reducing brain inflammation by directing microglia after chronic cerebral hypoperfusion to M2 polarization [47]. FTY720 has been reported to suppress TNF*α* and IL6 production in RAW264.7 macrophages [48]. The results of the present study are in agreement with these previous reports.

This is the first report showing that local FTY720 treatment improves mechanical stress-induced scarring in mice. Given that mechanical stress is a promoter of abnormal scarring in humans as well [3], it is possible that topical application of FTY720 will also effectively suppress human scarring. FTY720 is a prodrug that acts as an immunomodulator after activation [12]. It was discovered when myriocin (ISP-1), which is a metabolite of the fungus *Isaria sinclairii*, was chemically modified. Later, FTY720 was found to be a structural analog of S1P and a functional antagonist of S1PRs [49]. Unlike conventional immunosuppressive agents, FTY720 does not impair cellular DNA synthesis [50]. Our study suggests that topical application of local inflammatory lesion not only reduces macrophage numbers in the lesion but also inhibits vasculogenesis. Thus, FTY720 acts with a unique mechanism that differs from that of other immunosuppressive agents and is therefore likely to be suitable for topical application. These observations overall suggest that FTY720 may have clinical benefits in severe abnormal scar management, which is a field that has not progressed significantly for many years.

Further studies that identify the optimal safe and effective dosage and treatment regimen for local FTY720 injections are needed. In addition, more advanced investigations into the tissue and serum levels of FTY720 and FTY720-P would also be useful. We used a small dose that was applied three times (on Day 8, 10, and 12) after wounding. Despite the small dose, this regimen induced lymphocytopenia. This systemic immunosuppression is also an effect of topical corticosteroid injections [51]. Thus, the local treatment regimen for FTY720 should involve intervals of at least a month or longer; similar intervals are currently used when human abnormal scars are treated with corticosteroid injections [6].

Although our understanding of scar biology is improving, the wound healing process is complex and the mechanisms that lead to abnormal scar development remain unclear [52]. It is likely that the most effective treatment for abnormal scars is one that simultaneously focuses on one or several of multiple targets, particularly molecules and processes that are involved in inflammation, vasculogenesis, and fibrosis. Targeting inflammation and/or vasculogenesis is likely to be particularly effective when the treatment is applied prophylactically, namely, at the earliest stages of scar formation. However, for lesions like keloids that exhibit more advanced fibrosis, a combination of treatments for fibrosis may be necessary. In the future, further study on how FTY720 shows the effects beyond corticosteroids in developed human abnormal scars should be included. Since FTY720 simultaneously suppresses both inflammation and vasculogenesis, it can be a novel therapeutic option for treating severe abnormal scars. Topical application of FTY720 is likely to be effective for other inflammatory and allergic diseases as well.

## Data Availability

The data used to support the findings of this study are available from the corresponding author upon request.
